# Regulation of inflammatory arthritis by the upstream kinase mitogen activated protein kinase kinase 7 in the c-Jun N-Terminal kinase pathway

**DOI:** 10.1186/ar3750

**Published:** 2012-02-21

**Authors:** Sang-il Lee, David L Boyle, Andres Berdeja, Gary S Firestein

**Affiliations:** 1 Division of Rheumatology, Allergy and Immunology, UCSD School of Medicine, La Jolla, CA, USA; 2 Department of Internal Medicine and Institute of Health Science, Gyeongsang National University School of Medicine, Jinju, Republic of Korea; 3 Isis Pharmaceuticals, Inc., Carlsbad, CA, USA

**Keywords:** C-Jun N-terminal kinase, Mitogen-activated protein kinase kinase 7, Rheumatoid arthritis, Anti-sense oligonucleotide

## Abstract

**Introduction:**

The c-Jun N-terminal kinase (JNK) is a key regulator of matrix metalloproteinase (MMP) and cytokine production in rheumatoid arthritis (RA) and JNK deficiency markedly protects mice in animal models of arthritis. Cytokine-induced JNK activation is strictly dependent on the mitogen-activated protein kinase kinase 7 (MKK7) in fibroblast-like synoviocytes (FLS). Therefore, we evaluated whether targeting MKK7 using anti-sense oligonucleotides (ASO) would decrease JNK activation and severity in K/BxN serum transfer arthritis.

**Methods:**

Three 2'-O-methoxyethyl chimeric ASOs for MKK7 and control ASO were injected intravenously in normal C57BL/6 mice. PBS, control ASO or MKK7 ASO was injected from Day -8 to Day 10 in the passive K/BxN model. Ankle histology was evaluated using a semi-quantitative scoring system. Expression of MKK7 and JNK pathways was evaluated by quantitative PCR and Western blot analysis.

**Results:**

MKK7 ASO decreased MKK7 mRNA and protein levels in ankles by about 40% in normal mice within three days. There was no effect of control ASO on MKK7 expression and MKK7 ASO did not affect MKK3, MKK4 or MKK6. Mice injected with MKK7 ASO had significantly less severe arthritis compared with control ASO (*P *< 0.01). Histologic evidence of synovial inflammation, bone erosion and cartilage damage was reduced in MKK7 ASO-treated mice (*P *< 0.01). MKK7 deficiency decreased phospho-JNK and phospho-c-Jun in ankle extracts (*P *< 0.05), but not phospho-MKK4. Interleukin-1beta (IL-1β), MMP3 and MMP13 gene expression in ankle joints were decreased by MKK7 ASO (*P *< 0.01).

**Conclusions:**

MKK7 plays a critical regulatory role in the JNK pathway in a murine model of arthritis. Targeting MKK7 rather than JNK could provide site and event specificity when treating synovitis.

## Introduction

Rheumatoid arthritis (RA) is one of the most common immune-mediated diseases and is characterized by synovial inflammation and joint destruction [1]. Mitogen-activated protein kinases (MAPKs) are highly activated in rheumatoid synovium and potentially contribute to inflammatory and destructive mechanisms [2,3]. The c-Jun N-terminal kinases (JNKs), which belong to the MAPK family, play important roles in cytokine production and extracellular matrix degradation by regulating matrix metalloproteinase (MMP) in fibroblast-like synoviocytes (FLS) and animal models of RA [4,5]. Of the three JNK isoforms, JNK1 has been implicated as a pivotal regulator of synovial inflammation in murine arthritis due to its role in mast cell degranulation and macrophage migration [6,7].

JNK is activated via dual phosphorylation by two upstream MAPK kinases (MKKs), MKK4 and MKK7 [8-10]. The mice lacking MKK4 or MKK7 are embryonic lethal suggesting the two kinases are non-redundant and serve distinct functions [11]. Some studies suggest that these differences might be due to selective regulation by extracellular stimuli, distinct tissue distribution and different biochemical properties [10]. Thus, an alternative approach targeting the MKKs instead of JNK could suppress signaling responses that contribute to inflammatory arthritis but spare a subset of host defense or homoeostasis pathways.

Our previous studies showed that MKK4 and MKK7 are expressed and phosphorylated in RA synovium and both are activated by cytokines in RA FLS [12]. Surprisingly, cytokine-induced JNK activation and MMP production are strictly dependent on MKK7 in cytokine-stimulated FLS and do not require MKK4 [13]. Therefore, we evaluated whether selective targeting of MKK7 using anti-sense oligonucleotides (ASOs) would block arthritis-associated JNK activation and decreased arthritis severity in K/BxN serum transfer arthritis. The data indicate that blockade MKK7 mimics the effect of JNK deficiency and suppresses inflammatory arthritis.

## Materials and methods

### Oligonucleotides

A series of uniform chimeric 20-mer phosphorothioate oligonucleotides containing 2'-O-methoxyethyl chimeric (2'-MOE) groups at positions 1 to 5 and 15 to 20 targeted to murine MKK7 were synthesized and purified as described (kind gift of Isis Pharmaceuticals, Inc. (Carlsbad, CA, USA) [14]. Three ASOs complementary to murine MKK7 (Gen-Bank accession number AB005654) were 5'-TCTCCTGCAGCTTCTGGTCA-3', 5'-ACTTTGGTCTCTTCCTGTGA-3' and 5'-CCGTTCACAGTGTCTGTCGG-3'. The sequence for control ASO was 5'-CCTTCCCTGAAGGTTCCTCC-3'.

### ASO treatment in normal mice

All animal protocols received prior approval by the institutional review board. Pathogen-free male C57BL/6 mice were purchased from The Jackson Laboratory (Bar Harbor, ME, USA) and MKK7 or control ASOs were administered to mice based upon body weight by intravenous injection (25 and 50 mg/kg). Three days after injection of ASOs, mice were sacrificed and various tissues evaluated for MKK7 gene expression.

### K/BxN serum transfer arthritis and ASO treatment

To induce K/BxN serum transfer arthritis [15], serum samples were pooled from arthritic adult K/BxN mice and injected intraperitoneally (IP) as previously described [16]. C57BL/6 mice received PBS, MKK7 ASOs or control ASO (50 mg/kg) i.v. twice a week beginning on Day -8 and then administered 100 μl of K/BxN serum on Day 0. Clinical arthritis scores were evaluated using a scale of 0 to 4 for each paw for a total score of 16. Ankle thickness was measured with a caliper placed across the ankle joint at the widest point. Histopathologic assessment was performed using a semi-quantitative scoring system as previously described [6], including synovial inflammation, bone erosion and cartilage damage.

### Quantitative real time PCR

Ankle joints were collected at study termination, dissected to remove extra-articular tissue and snap-frozen in liquid nitrogen. The specimens were pulverized and total RNA was isolated using Rneasy^® ^Lipid Tissue kit per manufacturer's protocol (Qiagen, Valencia, CA, USA). MKK7, IL-1β, MMP3 and MMP13 expressions were measured by quantitative real-time PCR as previously described [17]. The threshold cycle (Ct) values were normalized to hypoxanthine-guanine phosphoribosyl transferase (HPRT) or glyceraldehyde-3-phosphate dehydrogenase (GAPDH) expression.

### Western blot analysis

Snap-frozen joints were pulverized and homogenized at 100 mg of tissue per 0.5 ml of lysis buffer. Western blot analysis was then performed as described previously [18]. Anti-MKK3, anti-MKK6 and anti-GAPDH antibodies were purchased from Santa Cruz Biotechnology (Santa Cruz, CA, USA). Anti-MKK4, anti-MKK7, anti-phospho-MKK4, anti-JNK, anti-phospho-JNK, anti-c-Jun and anti-phospho-c-Jun antibodies were purchased from Cell Signaling Technology (Danvers, MA, USA). Immunoreactive protein was detected with Immun-Star Western C kit (Bio-Rad, Hercules, CA, USA) using VersaDoc MP4000 imaging system (Bio-Rad). Densitometry analysis was carried out with Quantity One 1-D analysis software (Bio-Rad).

### Statistical analysis

Data are expressed as mean ± SE. Arthritis scores and change of ankle thickness among PBS, control ASO or MKK7 ASO-injected groups were analyzed by one-way ANOVA and Tukey's *post-hoc *test. Comparisons between control ASO and MKK7 ASO-injected groups were analyzed by two-tailed Student's *t*-test. In all tests, *P-*value < 0.05 was considered statistically significant.

## Results

### MKK7 knockdown by ASO in normal C57BL/6 mice

**Figure 1:**
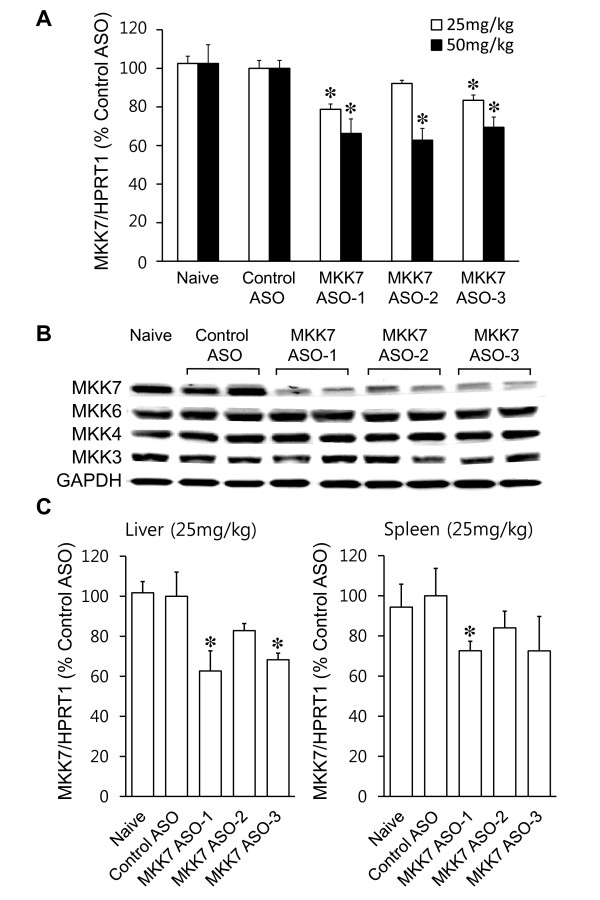
**MKK7 ASOs selectively reduce MKK7 mRNA and protein levels in normal C57BL/6 mice**. Three 2'-O-Methoxyethyl chimeric ASOs for MKK7 or control ASO were injected i.v. at the indicated doses and ankle joints, liver, and spleen were collected three days after injection of ASO. **(A) **Total mRNA was prepared from ankle joints and MKK7 mRNA expression was analyzed by quantitative PCR (Values = Mean ± SE, *n *= 3 mice for each group, *P < 0.05 vs. control ASO). **(B) **Western blot analysis of proteins in ankle lysates derived from naive and control or MKK7 ASO (50 mg/kg) injected mice. MKK7 ASOs decreased MKK7 protein levels but did not decrease MKK3, MKK4, and MKK6 levels. **(C) **MKK7 ASOs decreased MKK7 mRNA levels in liver and spleen (Values = Mean ± SE, *n *= 3 mice for each group, *P < 0.05 vs. control ASO).

Three different 2'-MOE chimeric ASOs (MKK7 ASO-1, -2, -3) targeting distinct regions of the MKK7 gene or control ASO were injected i.v. into normal C57BL/6 mice. Three days later ankle joints were harvested and assayed for MKK7 mRNA. As shown in Figure 1A, MKK7 mRNA levels were reduced in a dose-dependent manner (Figure 1A), with greatest inhibition at a dose of 50 mg/kg of MKK7 ASO-2 compared with control ASO (*P *< 0.05, *n *= 3 mice for each group). Control ASO had no effect. MKK7 protein levels in the ankle were also analyzed by Western blot analysis. Figure 1B shows that MKK7 protein levels were also decreased by MKK7 ASO, but MKK3, MKK4 and MKK6 levels were not affected. MKK7 mRNA was also decreased in liver and spleen by up to 37% at a dose of 25 mg/kg of MKK7 ASO-1 (Figure 1C; *P *< 0.05, *n *= 3 mice for each group) and maximally decreased in liver by up to 45% at a dose of 50 mg/kg of MKK7 ASO-1 (data not shown; *P *< 0.05).

### Effect of MKK7 ASO on K/BxN serum transfer arthritis

**Figure 2:**
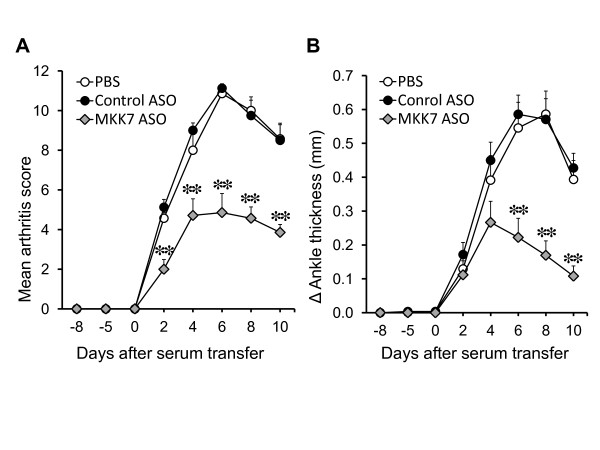
**MKK7 ASO suppresses the arthritis severity in K/BxN serum transfer arthritis**. K/BxN serum was injected on Day 0 and PBS, control or MKK7 ASO (50 mg/kg) were injected i.v. twice a week from Day -8. Arthritis severity was determined with semi-quantitative clinical scoring and change in ankle thickness. Values = Mean ± SE, *n *= 7 to 8 mice for each group,*P < 0.05, **P < 0.01 vs. control ASO.

MKK7 ASO-2 (hereafter called "MKK7 ASO") was selected for further *in vivo *experiments in passive K/BxN arthritis. C57BL/6 mice injected i.v. with PBS, MKK7 ASO or control ASO (50 mg/kg) twice a week beginning Day -8 and then administered K/BxN serum on Day 0. Mice injected with MKK7 ASO had less severe arthritis from Day 4 to Day 10 compared with control ASO (Figure 2). The peak clinical scores were 11.1 ± 0.2 in control ASO, 4.9 ± 1.0 in MKK7 ASO (*P *< 0.01) and the peak change in ankle diameter was 0.59 ± 0.06 mm in control ASO and 0.22 ± 0.06 mm in MKK7 ASO (*P *< 0.01).

### Effect of MKK7 ASO on histopathology

**Figure 3:**
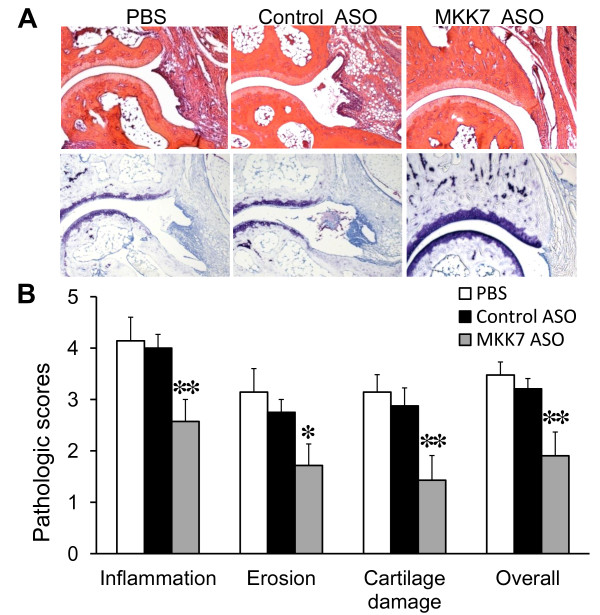
**MKK7 ASO reduces histologic damage in K/BxN serum transfer arthritis**. Hind paws were collected at Day 10 after treatment with MKK7 ASO and sectioned for histologic assessment as described. **(A) **Representative sections of the ankle joints stained with H&E (upper, ×100) and toludine blue (lower, ×100). **(B) **Histology scores in ankle joints. Values = Mean ± SE, *n *= 7 to 8 for each group, *P < 0.05 and **P < 0.01 vs. control ASO.

Histopathologic analysis was performed on ankle joints obtained on Day 10 after K/BxN serum administration. Consistent with the decreased clinical arthritis, MKK7 ASO suppressed synovial inflammation, bone erosions and cartilage destruction compared with control ASO (see Figure 3).

### Effect of MKK7 ASO on MKK4, JNK, and c-Jun phosphorylation

**Figure 4:**
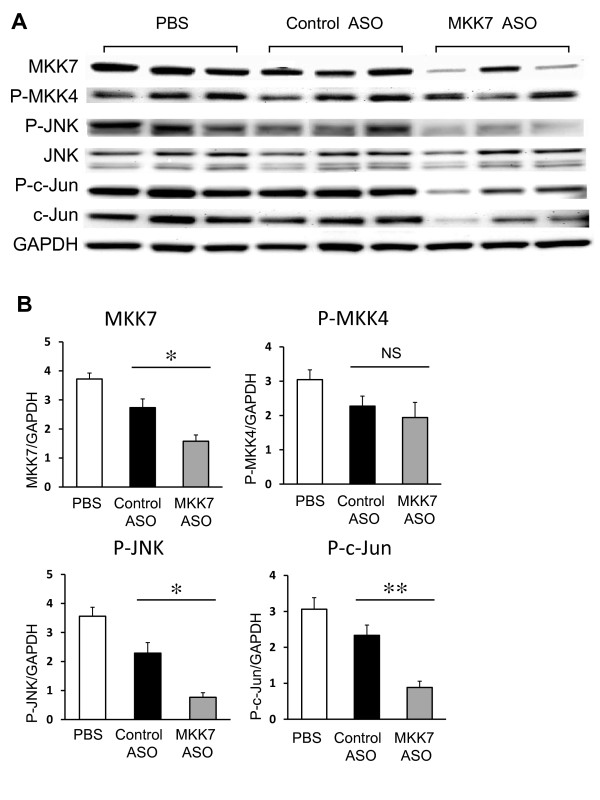
**MKK7 ASO reduces kinase phosphorylation in K/BxN serum transfer arthritis**. Ankle joints were collected at Day 10 after treatment with MKK7 ASO and homogenized as described. Western blot analysis was used to determine relative phosphorylation of MKK4, JNK, and c-Jun. **(A) **Representative experiment showing total and phosphorylated proteins. **(B) **Quantitative analysis of Western blot analysis after normalizing results to GAPDH. Values = Mean ± SE, *n *= 3 for each group, *P < 0.05 vs. control ASO.

Cytokine-induced JNK activation is dependent on MKK7 in cultured FLS and does not require MKK4. To determine the effect of selective MKK7 deficiency on JNK signaling *in vivo*, the ankle joints were evaluated by Western blot analysis to determine the phosphorylation state of MKK4, JNK and c-Jun. Consistent with the reduction of MKK7 protein level (42.2%, *P *< 0.05), MKK7 deficiency decreased GAPDH-normalized phospho-JNK by 67% and phospho-c-Jun by 62% compared with control ASO injected mice (*P *< 0.05, *n *= 3 for each group) (Figure 4). However, there was no significant difference of phosphorylation status of MKK4 between MKK7 ASO and control ASO injected groups. Similar results were obtained if the phospho-MKK4 and phospho-JNK were normalized to MKK4 and JNK, respectively. The c-Jun protein levels were higher in the control ASO-treated mice compared with MKK7 treatment due to increased local cytokine production, such as IL-1β (see below). Thus, normalization to GAPDH provides a more reliable assessment of total phospho-c-Jun in the tissue.

### Regulation of IL-1β and MMP expression by MKK7 deficiency

**Figure 5:**
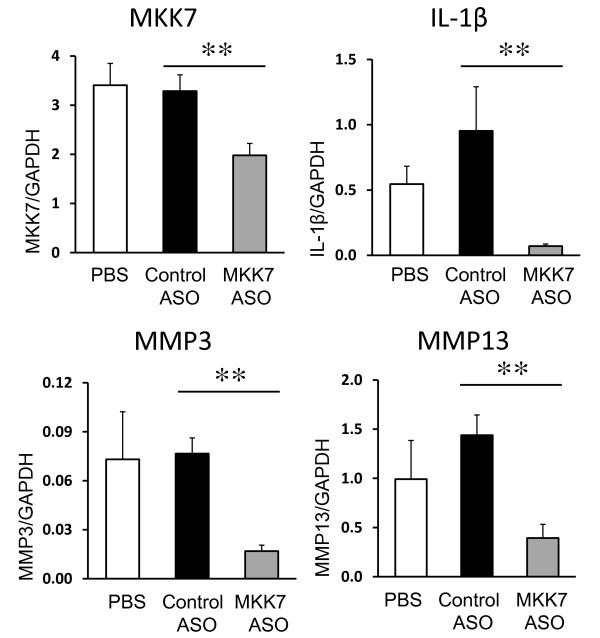
**MKK7 ASO reduces gene expression in K/BxN serum transfer arthritis**. Ankle joints were collected at day 10 after treatment with MKK7 ASO and analyzed using qPCR as described. Values = Mean ± SE, *n *= 7 to 8 for each group, **P < 0.01 vs. control ASO.

The JNK pathway regulates MMP gene expression. Consistent with the reduction phospho-JNK and phospho-c-Jun in ankle joints, MMP3 and MMP13 expression were significantly decreased in the mice injected with MKK7 ASO compared with control ASO (77.9%, and 72.6%, respectively, *P *< 0.05) (Figure 5). Of interest, IL-1β expression was also decreased. These data suggest that MKK7 plays a key role in regulating the JNK pathway, including transcription of inflammatory cytokines and proteases involved in joint damage.

## Discussion

Proinflammatory cytokines and MMPs promote synovial inflammation and facilitate cartilage and bone destruction in RA [5]. The MAPKs (ERK, JNK and p38) contribute by phosphorylating key transcription factors, such as activator protein-1 (AP-1), that are required for gene transcription. JNK, in particular, plays a pivotal role in cytokine-mediated AP-1 induction and MMP gene expression in FLS [4,19]. Three isoforms of JNK have been characterized, namely JNK1, 2 and 3. JNK1 and 2 are ubiquitous while JNK3 is primarily restricted to neurologic tissue [20]. JNK2 deficiency has only modest effects in pre-clinical models of arthritis, but JNK1 deficiency attenuates synovitis and joint destruction in murine antigen-induced arthritis and passive K/BxN serum transfer arthritis [6,7,21]. JNK1 also contributes to osteoclast differentiation, since JNK1-deficient osteoclast progenitors do not mature into bone-resorbing osteoclasts [22]. These data suggest that JNK participates in the synovial inflammation and joint destruction of RA and could potentially be targeted in diseases like RA.

While JNKs are attractive targets, they regulate in many normal cell functions, especially in matrix remodeling and host defense [23,24]. Thus, blocking all JNK activity, or even all JNK1 activity, could affect host defense or matrix homeostasis. As an alternative strategy, targeting an individual upstream kinase like MKK4 or MKK7 could permit some normal JNK functions while interfering with a subset that is pathogenic in synovitis. MKK4 and MKK7, two JNK upstream kinases, exhibit some different properties although they can synergistically activate JNKs [10]. TNF and IL-1 mainly activate MKK7 in murine embryonic fibroblasts, while ultraviolet radiation, anisomycin, heat and osmotic shock activate both MKK4 and MKK7 [8,9,25]. These data suggest that MKK4 and MKK7 contribute separately to the activation of JNKs in response to environmental stress or inflammatory cytokines.

We previously showed that MKK7, but not MKK4, is required for IL-1-induced JNK phosphorylation and AP-1-driven MMP expression [13]. Nevertheless, MKK4 is a component of the JNK signal complex and is also readily phosphorylated in FLS [12]. Mice lacking Gadd45β, which serves as an endogenous inhibitor of MKK7, have enhanced JNK activity and disease severity in the passive K/BxN model [16]. These data suggest that selective MKK7 blockade could suppress arthritis and potentially decrease adverse effects by permitting non-pathogenic MKK4-mediated JNK activation. However, there is no direct evidence that MKK7 inhibition would be beneficial in synovitis. Our initial plans to focus on Gadd45β were complicated by the recent observation that Gadd45β deficiency unexpectedly exacerbates disease severity in collagen-induced arthritis [26].

We, therefore, focused on genetic approaches that circumvent the embryonic lethality of MKK7 deficiency. Several small interfering RNA (siRNA) methods were tested because others have reported success [27], but we were unable to consistently knockdown endogenous MKK7 expression (data not shown). Chemically modified ASOs were then tested for applications in animal models of RA because of their nuclease-resistant capacity, potency and long half-life [28-30]. Free ASOs are considerably smaller than siRNA-delivery agent complexes and enter many cells types via pinocytosis and phagocytosis, whereas larger siRNA complexes primarily enter macrophages and neutrophils by phagocytosis [31,32]. Thus, we used single strand, 2'-O-methoxyethylribose modified chimeric ASOs to investigate the effect of MKK7 deficiency in mice. Selectivity was confirmed with MKK7 ASOs, which decreased MKK7 mRNA and protein expression but not MKK3, MKK4 or MKK6.

The ASO studies showed that selective MKK7 deficiency significantly reduced arthritis severity and joint destruction compared with control ASO-injected group even though MKK7 was only partially depleted. Downstream events were consistent with previous *in vitro *studies by demonstrating reduced phosphorylation of JNK and c-Jun in the inflamed joints of MKK7 ASO-treated mice. Decreased joint damage in mice treated with MKK7 ASOs is consistent with previous observations that MKK7 is a pivotal signaling molecule that regulates JNK and MMP expression in FLS [13].

Taken together, these results imply that MKK7 plays a pivotal role in inflammatory arthritis and that MKK7 ASO acts through the inhibition of JNK in passive K/BxN arthritis. Because JNK2 does not contribute to this model, the effect is most likely due to decreased JNK1 activation with resultant decreased mast cell activation [6]. That observation is supported by the fact that JNK activation is abolished in *mkk7*^-/- ^mast cell lines, suggesting that MKK7 is essential for JNK activation in mast cells [33].

## Conclusion

MKK7 plays a critical role in JNK pathway *in vivo*, and MKK7 deficiency suppresses arthritis severity and joint destruction. Selective MKK7 inhibition represents a promising alternative approach to blocking downstream kinases directly. This strategy is consistent with recent successes targeting upstream kinases like spleen tyrosine kinase (Syk) and Janus kinase (JAK) in RA and suggests that targeting upstream kinases might be useful for RA [34]

## Abbreviations

AP-1: activator protein-1; ASO: anti-sense oligonucleotides; Ct: threshold cycle; ERK: extra-cellular signal regulated kinase; FLS: fibroblast-like synoviocytes; GAPDH: glyceraldehyde-3-phosphate dehydrogenase; HPRT: hypoxanthine-guanine phosphoribosyl transferase; IL: interleukin; IP: intraperitoneally; JAK: Janus kinase; JNK: c-Jun N-Terminal Kinase; MAPK: mitogen activated protein kinase; MKK7: mitogen activated protein kinase kinase 7; MMP: matrix metalloproteinase; RA: rheumatoid arthritis; Syk: spleen tyrosine kinase.

## Competing interests

Dr. Berdeja is an employee of Isis Pharmaceuticals, Inc. There are no other competing interests.

## Authors' contributions

Each author took part in this paper and its publication is approved by all authors. SL performed all experiments, generated the figures and wrote the draft manuscript. DB participated in its design and coordination, and helped with experiments. AB performed synthesis and purifying of antisense oligomers. GSF designed, organized and analyzed the data, and wrote the manuscript.

## References

[B1] Firestein GS Evolving concepts of rheumatoid arthritis Nature 2003 423 356 361 10.1038/nature01661 12748655

[B2] Fukushima A Boyle DL Corr M Firestein GS Kinetic analysis of synovial signalling and gene expression in animal models of arthritis Ann Rheum Dis 2010 69 918 923 10.1136/ard.2009.112201 19473996 PMC2859101

[B3] Thalhamer T McGrath MA Harnett MM MAPKs and their relevance to arthritis and inflammation Rheumatology (Oxford) 2008 47 409 414 18187523 10.1093/rheumatology/kem297

[B4] Han Z Boyle DL Aupperle KR Bennett B Manning AM Firestein GS Jun N-terminal kinase in rheumatoid arthritis J Pharmacol Exp Ther 1999 291 124 130 10490895

[B5] Han Z Boyle DL Chang L Bennett B Karin M Yang L Manning AM Firestein GS c-Jun N-terminal kinase is required for metalloproteinase expression and joint destruction in inflammatory arthritis J Clin Invest 2001 108 73 81 11435459 10.1172/JCI12466 PMC209341

[B6] Guma M Kashiwakura J Crain B Kawakami Y Beutler B Firestein GS Kawakami T Karin M Corr M JNK1 controls mast cell degranulation and IL-1{beta} production in inflammatory arthritis Proc Natl Acad Sci USA 2010 107 22122 22127 10.1073/pnas.1016401107 21135226 PMC3009768

[B7] Guma M Ronacher LM Firestein GS Karin M Corr M JNK-1 deficiency limits macrophage-mediated antigen-induced arthritis Arthritis Rheum 2011 63 1603 1612 10.1002/art.30271 21305529 PMC3106119

[B8] Fleming Y Armstrong CG Morrice N Paterson A Goedert M Cohen P Synergistic activation of stress-activated protein kinase 1/c-Jun N-terminal kinase (SAPK1/JNK) isoforms by mitogen-activated protein kinase kinase 4 (MKK4) and MKK7 Biochem J 2000 352 145 154 10.1042/0264-6021:3520145 11062067 PMC1221441

[B9] Tournier C Dong C Turner TK Jones SN Flavell RA Davis RJ MKK7 is an essential component of the JNK signal transduction pathway activated by proinflammatory cytokines Genes Dev 2001 15 1419 1426 10.1101/gad.888501 11390361 PMC312702

[B10] Wang X Destrument A Tournier C Physiological roles of MKK4 and MKK7: insights from animal models Biochim Biophys Acta 2007 1773 1349 1357 10.1016/j.bbamcr.2006.10.016 17157936

[B11] Asaoka Y Nishina H Diverse physiological functions of MKK4 and MKK7 during early embryogenesis J Biochem 2010 148 393 401 20801953 10.1093/jb/mvq098

[B12] Sundarrajan M Boyle DL Chabaud-Riou M Hammaker D Firestein GS Expression of the MAPK kinases MKK-4 and MKK-7 in rheumatoid arthritis and their role as key regulators of JNK Arthritis Rheum 2003 48 2450 2460 10.1002/art.11228 13130464

[B13] Inoue T Hammaker D Boyle DL Firestein GS Regulation of JNK by MKK-7 in fibroblast-like synoviocytes Arthritis Rheum 2006 54 2127 2135 10.1002/art.21919 16802349

[B14] Baker BF Lot SS Condon TP Cheng-Flournoy S Lesnik EA Sasmor HM Bennett CF 2'-O-(2-Methoxy)ethyl-modified anti-intercellular adhesion molecule 1 (ICAM-1) oligonucleotides selectively increase the ICAM-1 mRNA level and inhibit formation of the ICAM-1 translation initiation complex in human umbilical vein endothelial cells J Biol Chem 1997 272 11994 12000 10.1074/jbc.272.18.11994 9115264

[B15] Kouskoff V Korganow AS Duchatelle V Degott C Benoist C Mathis D Organ-specific disease provoked by systemic autoimmunity Cell 1996 87 811 822 10.1016/S0092-8674(00)81989-3 8945509

[B16] Svensson CI Inoue T Hammaker D Fukushima A Papa S Franzoso G Schett G Corr M Boyle DL Firestein GS Gadd45beta deficiency in rheumatoid arthritis: enhanced synovitis through JNK signaling Arthritis Rheum 2009 60 3229 3240 10.1002/art.24887 19877043 PMC2858378

[B17] Boyle DL Rosengren S Bugbee W Kavanaugh A Firestein GS Quantitative biomarker analysis of synovial gene expression by real-time PCR Arthritis Res Ther 2003 5 R352 360 10.1186/ar1004 14680510 PMC333415

[B18] Hammaker DR Boyle DL Inoue T Firestein GS Regulation of the JNK pathway by TGF-beta activated kinase 1 in rheumatoid arthritis synoviocytes Arthritis Res Ther 2007 9 R57 10.1186/ar2215 17559674 PMC2206340

[B19] Liacini A Sylvester J Li WQ Zafarullah M Inhibition of interleukin-1-stimulated MAP kinases, activating protein-1 (AP-1) and nuclear factor kappa B (NF-kappa B) transcription factors down-regulates matrix metalloproteinase gene expression in articular chondrocytes Matrix Biol 2002 21 251 262 10.1016/S0945-053X(02)00007-0 12009331

[B20] Davis RJ Signal transduction by the JNK group of MAP kinases Cell 2000 103 239 252 10.1016/S0092-8674(00)00116-1 11057897

[B21] Denninger K Rasmussen S Larsen JM Orskov C Seier Poulsen S Sorensen P Christensen JP Illges H Odum N Labuda T JNK1, but not JNK2, is required in two mechanistically distinct models of inflammatory arthritis Am J Pathol 2011 179 1884 1893 10.1016/j.ajpath.2011.06.019 21839715 PMC3181375

[B22] David JP Sabapathy K Hoffmann O Idarraga MH Wagner EF JNK1 modulates osteoclastogenesis through both c-Jun phosphorylation-dependent and -independent mechanisms J Cell Sci 2002 115 4317 4325 10.1242/jcs.00082 12376563

[B23] Nishina H Fischer KD Radvanyi L Shahinian A Hakem R Rubie EA Bernstein A Mak TW Woodgett JR Penninger JM Stress-signalling kinase Sek1 protects thymocytes from apoptosis mediated by CD95 and CD3 Nature 1997 385 350 353 10.1038/385350a0 9002521

[B24] Sabapathy K Jochum W Hochedlinger K Chang L Karin M Wagner EF Defective neural tube morphogenesis and altered apoptosis in the absence of both JNK1 and JNK2 Mech Dev 1999 89 115 124 10.1016/S0925-4773(99)00213-0 10559486

[B25] Moriguchi T Toyoshima F Masuyama N Hanafusa H Gotoh Y Nishida E A novel SAPK/JNK kinase, MKK7, stimulated by TNFalpha and cellular stresses EMBO J 1997 16 7045 7053 10.1093/emboj/16.23.7045 9384583 PMC1170307

[B26] Luo Y Boyle DL Hammaker D Edgar M Franzoso G Firestein GS Suppression of collagen-induced arthritis in growth arrest and DNA damage inducible 45beta (Gadd45beta)-deficient mice Arthritis Rheum 2011 63 2949 2955 10.1002/art.30497 21702006 PMC3183142

[B27] Courties G Presumey J Duroux-Richard I Jorgensen C Apparailly F RNA interference-based gene therapy for successful treatment of rheumatoid arthritis Expert Opin Biol Ther 2009 9 535 538 10.1517/14712590902926089 19392574

[B28] Crooke RM Graham MJ Lemonidis KM Whipple CP Koo S Perera RJ An apolipoprotein B antisense oligonucleotide lowers LDL cholesterol in hyperlipidemic mice without causing hepatic steatosis J Lipid Res 2005 46 872 884 10.1194/jlr.M400492-JLR200 15716585

[B29] Geary RS Watanabe TA Truong L Freier S Lesnik EA Sioufi NB Sasmor H Manoharan M Levin AA Pharmacokinetic properties of 2'-O-(2-methoxyethyl)-modified oligonucleotide analogs in rats J Pharmacol Exp Ther 2001 296 890 897 11181921

[B30] White PJ Anastasopoulos F Pouton CW Boyd BJ Overcoming biological barriers to *in vivo *efficacy of antisense oligonucleotides Expert Rev Mol Med 2009 11 e10 19302730 10.1017/S1462399409001021

[B31] Conner SD Schmid SL Regulated portals of entry into the cell Nature 2003 422 37 44 10.1038/nature01451 12621426

[B32] Huang L Sullenger B Juliano R The role of carrier size in the pharmacodynamics of antisense and siRNA oligonucleotides J Drug Target 2010 18 567 574 10.3109/10611861003734019 20367081

[B33] Sasaki T Wada T Kishimoto H Irie-Sasaki J Matsumoto G Goto T Yao Z Wakeham A Mak TW Suzuki A Cho SK Zuniga-Pflucker JC Oliveira-dos-Santos AJ Katada T Nishina H Penninger JM The stress kinase mitogen-activated protein kinase kinase (MKK)7 is a negative regulator of antigen receptor and growth factor receptor-induced proliferation in hematopoietic cells J Exp Med 2001 194 757 768 10.1084/jem.194.6.757 11560992 PMC2195963

[B34] Hammaker D Firestein GS "Go upstream, young man": lessons learned from the p38 saga Ann Rheum Dis 2010 69 Suppl 1 i77 82 10.1136/ard.2009.119479 19995751 PMC2911016

